# Palladium nanoparticles anchored to anatase TiO_2_ for enhanced surface plasmon resonance-stimulated, visible-light-driven photocatalytic activity

**DOI:** 10.3762/bjnano.6.43

**Published:** 2015-02-11

**Authors:** Kah Hon Leong, Hong Ye Chu, Shaliza Ibrahim, Pichiah Saravanan

**Affiliations:** 1 Environmental Engineering Laboratory, Department of Civil Engineering, Faculty of Engineering, University of Malaya, 50603, Kuala Lumpur, Malaysia; 2 Nanotechnology & Catalysis Research Center (NANOCAT), University of Malaya, 50603, Kuala Lumpur, Malaysia

**Keywords:** endocrine disrupting compound, nano photocatalysts, noble metal, photodeposition, sunlight

## Abstract

Freely assembled palladium nanoparticles (Pd NPs) on titania (TiO_2_) nano photocatalysts were successfully synthesized through a photodeposition method using natural sunlight. This synthesized heterogeneous photocatalyst (Pd/TiO_2_) was characterized through field emission scanning electron microscopy (FESEM), high resolution transmission electron microscopy (HRTEM), X-ray diffraction (XRD), BET surface area, UV–vis diffuse reflectance spectra (UV-DRS), Raman and photoluminescence (PL) analyses. The simple and smart synthesis anchored well the deposition with controlled Pd NPs size ranging between 17 and 29 nm onto the surface of TiO_2_. Thus, it gives the characteristic for Pd NPs to absorb light in the visible region obtained through localized surface plasmon resonance (LSPRs). Apparently, the photocatalytic activity of the prepared photocatalysts was evaluated by degrading the endocrine disrupting compound (EDC) amoxicillin (AMX) excited under an artificial visible light source. In the preliminary run, almost complete degradation (97.5%) was achieved in 5 h with 0.5 wt % Pd loading and the degradation followed pseudo-first-order kinetics. The reusability trend proved the photostability of the prepared photocatalysts. Hence, the study provides a new insight about the modification of TiO_2_ with noble metals in order to enhance the absorption in the visible-light region for superior photocatalytic performance.

## Introduction

Heterogeneous photocatalysts that employ TiO_2_ as metal oxide photocatalyst have raised the interest of many researchers since the discovery of the photocatalytic splitting of water under UV light irradiation by Fujishima and Honda in 1972 [1]. To date, TiO_2_ is still the most favorable choice owing to its versatility and robust advantages that include photostability, non-toxicity, low cost, chemical and biological inertness, high photocatalytic activity and favorable optoelectronic properties [2–5]. TiO_2_ also possesses an appropriate band gap that ensure the simultaneous formation of superoxide anions (•O_2_^−^) and hydroxyl (•OH) radicals for the oxidation of organic compounds [6]. Despite all these advantages, TiO_2_ has two major drawbacks, which are (1) a wide band gap (ca. 3.2 eV for anatase) that restricts the excitation strictly to UV light irradiation [7–10], and (2) a high recombination rate of photogenerated electron and hole pairs that leads to low photonic efficiency, which in turn hampers the photocatalytic reactions [7,11–12].

Numerous modifications have been reported by researchers to overcome the drawbacks of TiO_2_. This includes doping with either metallic/non-metallic species [13–16], forming heterostructures [17–18], and fine-tuning the morphology [19–22]. Alternatively, the coupling of semiconductor photocatalysts with noble metals (Au, Ag, Pd, Pt) turns out to be the most promising strategy to defeat the limitations of TiO_2_. This is due to the characteristics of noble metals, which can drastically enhance the absorption of visible light through localized surface plasmon resonance effects (LSPRs) [23–24]. The LSPR absorption in noble metal NPs arise from the collective oscillation of conduction electrons that are induced by the incident electromagnetic radiation [9]. Moreover, the formation of Schottky barriers caused by the contact of noble metal NPs with the semiconductor photocatalyst further enhance the separation of electrons and holes, which in turn reduce the electron and hole recombination rates drastically [5,10–11]. Ingram et al. have shown that the use of noble metals to synthesize Au/TiO_2_ and Ag/TiO_2_ NPs for water splitting increased absorption of visible light by a factor of 10 as compare to N-TiO_2_ [25]. Jiang et al. successfully fabricated one-dimensional anatase TiO_2_/Ag, which exhibited an excellent photocatalytic activity with almost 100% degradation of 2,4-dichlorophenol within 2 h [26]. Likewise, Hou et al. reported a 9-fold improvement in the photocatalytic decomposition rate of methyl orange driven by a photocatalyst consisting of robust plasmonic Au nanoparticles deposited on top of TiO_2_ [27]. While Mohapatra et al. had synthesized TiO_2_ nanotubes with palladium (Pd) NPs for the photocatalytic decomposition of azo dyes under sunlight irradiation. Pd/TiO_2_ nanotubes showed a faster degradation time (150 min) to completely decompose azo dye as compare to TiO_2_ nanotubes (250 min) [28]. Similarly, Kwak et al. found that by incorporating Pd into TiO_2_ led to an improved hydrogen production compared to pure TiO_2_ [29]. Hence, it is clear that the inclusion of noble metals either as dopant or composite contributes to an enhanced visible-light photoactivity.

There are several synthesis methods available for preparing plasmonic photocatalysts, namely photodeposition [3,30–31], hydrothermal [4,32–34], ion exchange [35–36], chemical reduction [25,37–38], physical vapour deposition [27,39–40], and deposition–precipitation [41–43]. Among these methods the photodeposition is the simplest. Most of the photodeposition employs artificial UV light as the source of energy. However, the utilization of sunlight instead of an artificial UV light source could be a sustainable alternative. The natural light effectively reduces the noble metal onto the TiO_2_ surface and promotes the application of a non-conventional energy resource. In the present study, a smart, easy and sustainable method for anchoring noble metal onto the surface of TiO_2_ is reported. Palladium was chosen as the noble metal for the study due to its high reactivity and reluctance toward surface oxidation [44–46]. The photocatalytic evaluation was studied by degrading the well-known antibiotic amoxicillin as model compound under irradiation with artificial visible light. To the best of our knowledge, this is the first report on utilizing the LSPR concept sustainably through Pd NPs onto anatase TiO_2_ for the enhancement of visible-light-driven photocatalysis.

## Results and Discussion

### Synthesis of Pd/TiO_2_ through solar-assisted photodeposition

**Figure 1:**
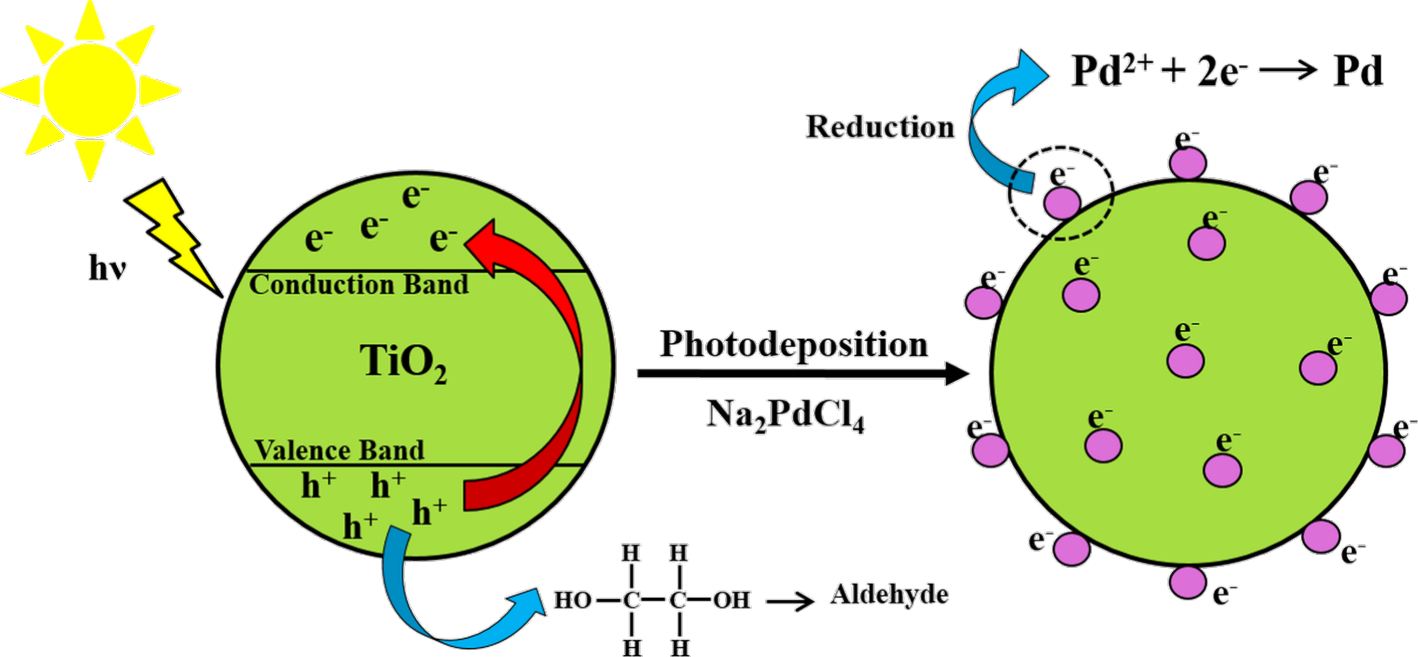
Schematic diagram for the synthesis of Pd/TiO_2_ trough solar-assisted photodeposition.

The Pd/TiO_2_ nanoparticles were synthesized through photodeposition using solar energy. The irradiation with sunlight was appropriate for palladium to be reduced and deposited onto the TiO_2_ surface and, thus, offered a sustainable synthesis route. This was achieved by exposing TiO_2_ to sunlight, through which free electrons and holes are generated. The electrons are excited into the conduction band (CB) which serves as an electron source for the eduction of palladium cations. The photoelectrons generated by TiO_2_ reduce the Pd^2+^ to palladium nanoparticles. Meanwhile, the photogenerated holes from the valence band (VB) react with ethylene glycol to form aldehyde. The complete mechanism is illustrated in Figure 1.

### Morphology and crystal structure

**Figure 2:**
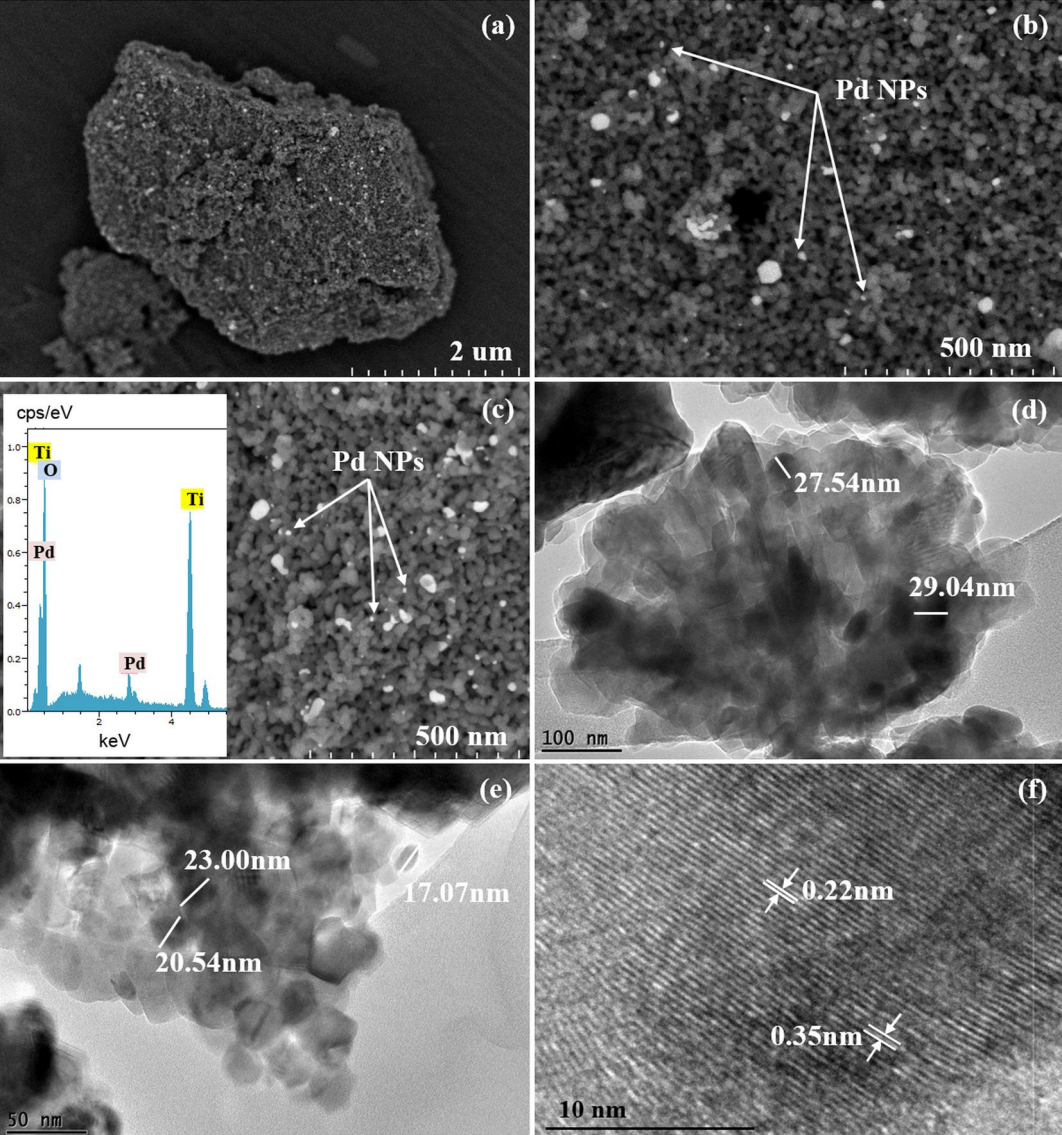
FESEM images of a) low magnification, b,c) high magnification of 0.5 wt % Pd/TiO_2_. The inset of c) is the EDX spectrum of 0.5 wt % Pd/TiO_2_ and d–f) HRTEM images of 0.5 wt % Pd/TiO_2_.

Figure 2a–c and Figure S1 (Supporting Information File 1) depict the FESEM images of successfully deposited Pd onto anatase TiO_2_. From Figure 2a, it is evident that the synthesized palladium NPs are uniformly deposited onto the surface of TiO_2_. It can be clearly seen from Figure 2b and Figure 2c that the TiO_2_ particles have a spheroid shape with a homogenous distribution. The Pd NPs exhibit a spherical morphology with particles size ranging from 17 to 29 nm. The inset in Figure 2c shows the corresponding EDX spectrum, confirming the presence of Ti, O and Pd in the synthesized Pd/TiO_2_ photocatalysts. The absence of chlorine from the EDX spectrum clearly indicates that the Cl^−^ ions from the titanium(IV) chloride (TiCl_4_) precursor was completely removed through an appropriate washing method thus eliminating the unwanted anion (Cl^-^) that suppress the photocatalytic activity. The HRTEM images (Figure 2d and Figure 2e) further confirm the formation of Pd/TiO_2_ without changing the original morphology of TiO_2_. It also further confirms the particles size of palladium. The lattice fringes with spacings of 0.22 and 0.35 nm as seen in Figure 2f can be clearly attributed to the Pd(111) and anatase TiO_2_(101) planes, respectively [28,47–49]. This clearly proves the formation of heterojunctions between Pd and TiO_2_.

**Figure 3:**
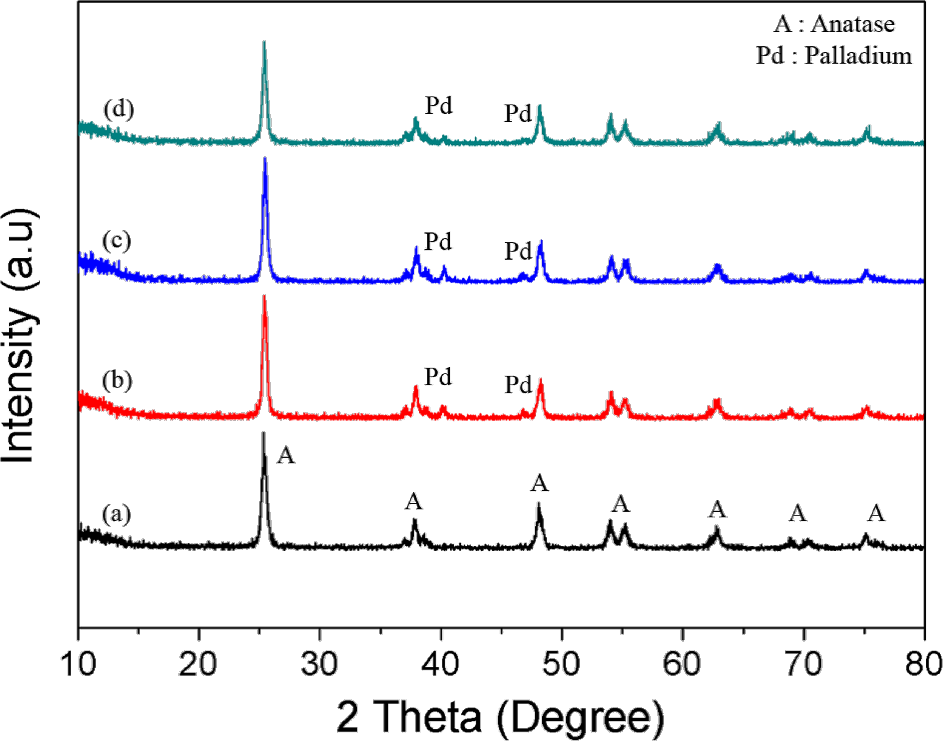
X-ray diffraction patterns of a) TiO_2_, b) 0.5 wt % Pd/TiO_2_, c) 1.0 wt % Pd/TiO_2_ and d) 3.0 wt % Pd/TiO_2_.

**Figure 4:**
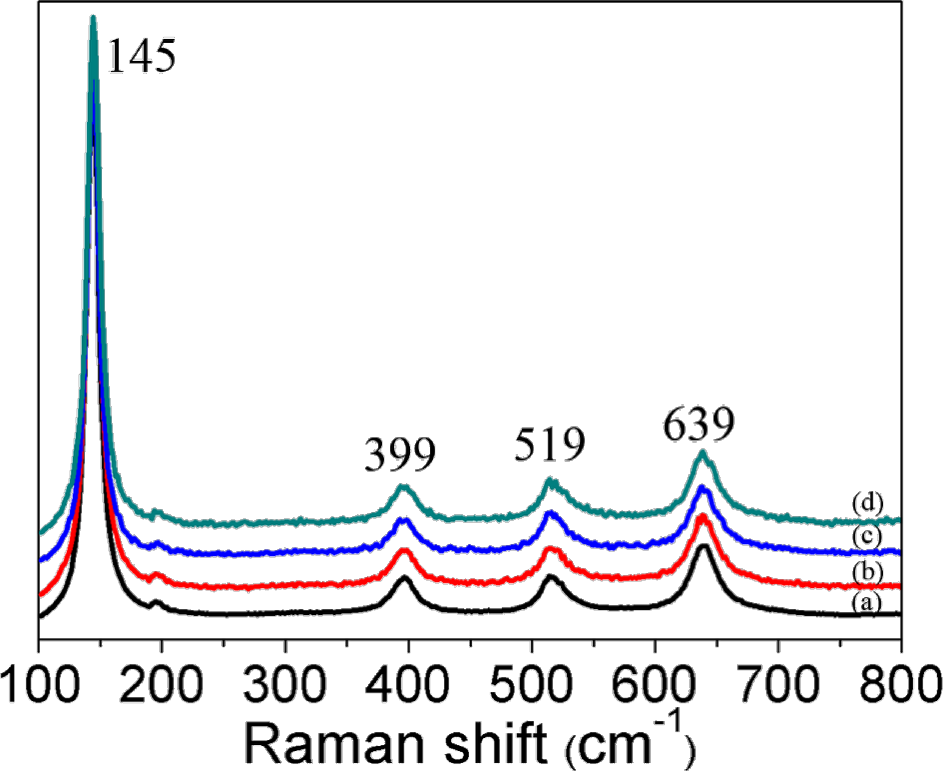
Raman spectra of a) TiO_2_, b) 0.5 wt % Pd/TiO_2_, c) 1.0 wt % Pd/TiO_2_ and d) 3.0 wt % Pd/TiO_2_.

Figure 3 shows the X-ray diffraction patterns of anatase TiO_2_ and Pd/TiO_2_ with different Pd loadings (0.5 wt %, 1.0 wt % and 3.0 wt %). Pure anatase TiO_2_ is observed in all samples indicating that its crystallinity is not affected by the solar-assisted photodeposition of Pd NPs. The diffraction patterns of the prepared anatase TiO_2_ correlated well with the standard peaks (JCPDS no. 21-1272) with the two most obvious diffraction peaks observed at 25.3° (101) and 48.0° (200). The obtained pattern also proved the absence of the rutile and brookite phases. The presence of Pd NPs are indicated by diffraction peaks appearing at 2θ values of 40.1 and 46.7°. They were assigned to the (111) and (200) crystal plane spacings of face centered cubic (FCC) Pd (JCPDS no 46-1043), respectively [50]. Only two peaks of three peaks that designated Pd were observed. This was because one peaks overlap with the anatase TiO_2_ at 2θ = 68.1° (220). Thus, these diffraction peaks further signify the metallic state of the loaded Pd NPs. The peaks also confirm the stability of the synthesized palladium crystals which may lead to promising visible light performance [51]. Overall, the prepared samples showed high crystallinity which is proven by the sharp peaks obtained from all samples. The average crystallite sizes of anatase TiO_2_ and Pd particles were calculated by using the Scherrer formula,

[1]
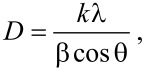


where *D* is the crystallite size (nm), *k* is a shape constant (in this case 0.9), λ is the wavelength of Cu Kα radiation (0.154 nm), θ is the diffraction angle (°) and β is the full width at half maximum. The crystallite size of the Pd particles were found to be 21.22 nm, 22.41 nm and 28.10 nm for 0.5 wt % Pd/TiO_2_, 1.0 wt % Pd/TiO_2_ and 3.0 wt % Pd/TiO_2_, respectively. No significant changes of the crystallite size of anatase TiO_2_ (19.75 nm) was observed after depositing Pd (19.57 nm). This clarified that Pd was deposited onto the TiO_2_ surface and was not incorporated into the TiO_2_ lattice. The Raman spectra of the synthesized samples are shown in Figure 4. Four distinct peaks were detected at 145 (E_g_), 399 (B_1g_), 519 (A_1g_ + B_1g_) and 639 cm^−1^ (E_g_) that attributes to the pristine anatase phase of the synthesized TiO_2_. These well matched with XRD analysis where the prepared samples are purely in crystalline anatase phase with the absence of band at 445 and 612 cm^−1^ corresponding to the rutile phase [52].

### BET surface area and XPS analysis

**Figure 5:**
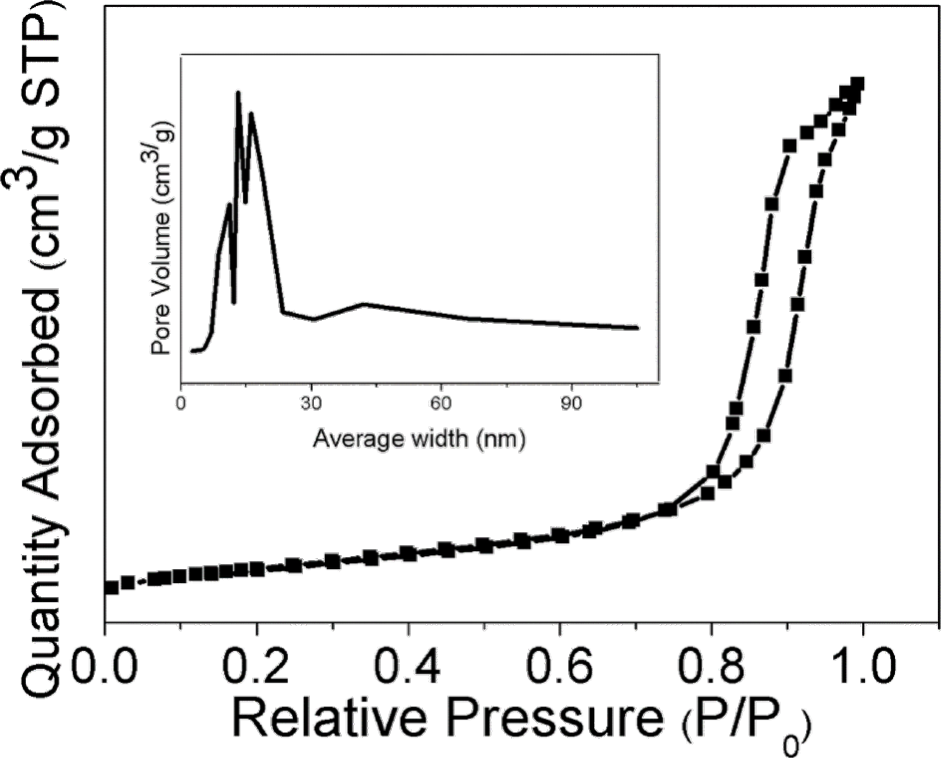
Adsorption–desorption isotherm of 0.5 wt % Pd/TiO_2_ and the inset is the pore size distribution.

**Figure 6:**
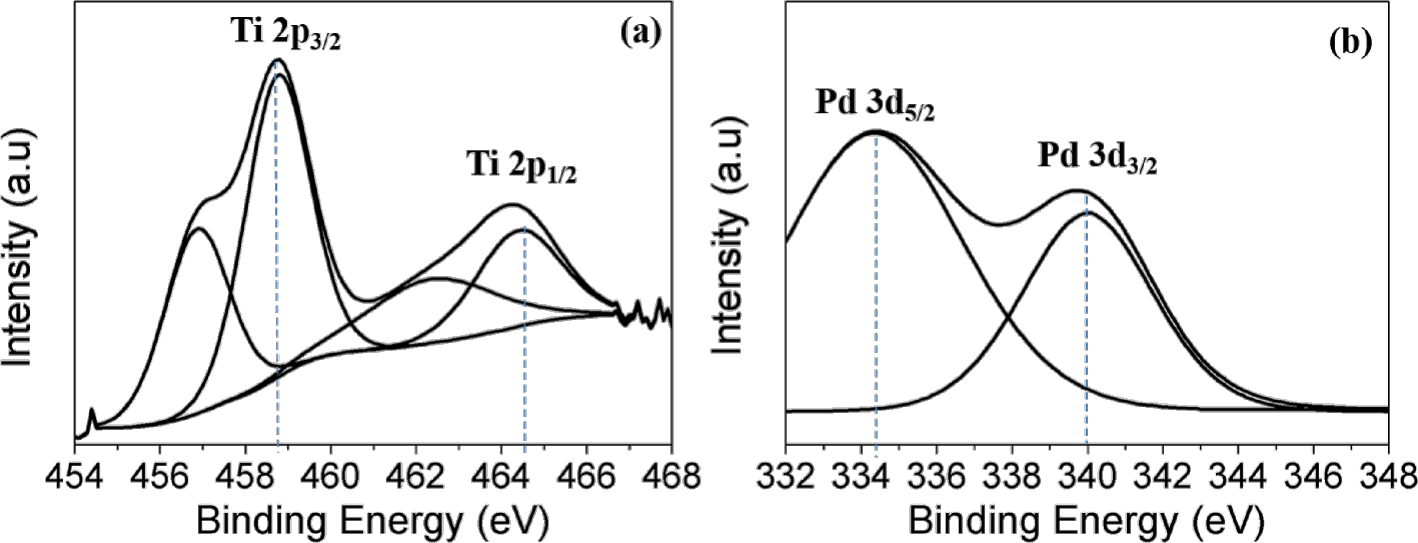
Core level XPS spectra of a) Ti 2p and b) Pd 3d of 0.5 wt % Pd/TiO_2_.

The nitrogen adsorption–desorption isotherms and corresponding pore size distribution of the prepared samples are depicted in Figure 5 and Figure S2 (Supporting Information File 1). As can be seen, all the samples possess a stepwise adsorption and desorption hysteresis, represented by type-IV isotherms, with the characteristics of a mesoporous material [53]. The variations in BET surface area, average pore size and pore volume after Pd deposition is summarized in Table S1 (Supporting Information File 1). In addition, the average pore diameter, determined through the Barrett–Joyner–Halenda (BJH) method using the desorption isotherm (inset of Figure 5 and Figure S2, Supporting Information File 1) was found to decrease after the deposition of Pd NPs. The decrease of BET surface area and average pore diameter was due to a minor blocking of the pores in anatase TiO_2_ by the deposited Pd NPs [48]. In order to determine the chemical composition and oxidation state of the prepared photocatalysts, X-ray photoelectron spectroscopy (XPS) was employed. As shown in Figure 6a, there are two peaks observed at binding energies of 458.8 eV and 464.4 eV, which correspond to Ti 2p_3/2_ and Ti 2p_1/2_ spin–orbit-splitting photoelectrons for pure anatase TiO_2_ [29]. These indicate the presence of typical Ti^4+^ in the synthesized samples. The presence of Pd NPs can be distinguished by two peaks centered at binding energies of 334.3 eV and 340.0 eV, which are assigned to Pd 3d_5/2_, and Pd 3d_3/2_, respectively (Figure 6b) [48] and confirm the predominantly metallic form of the deposited noble metal.

### Optical absorption and photoluminescence

**Figure 7:**
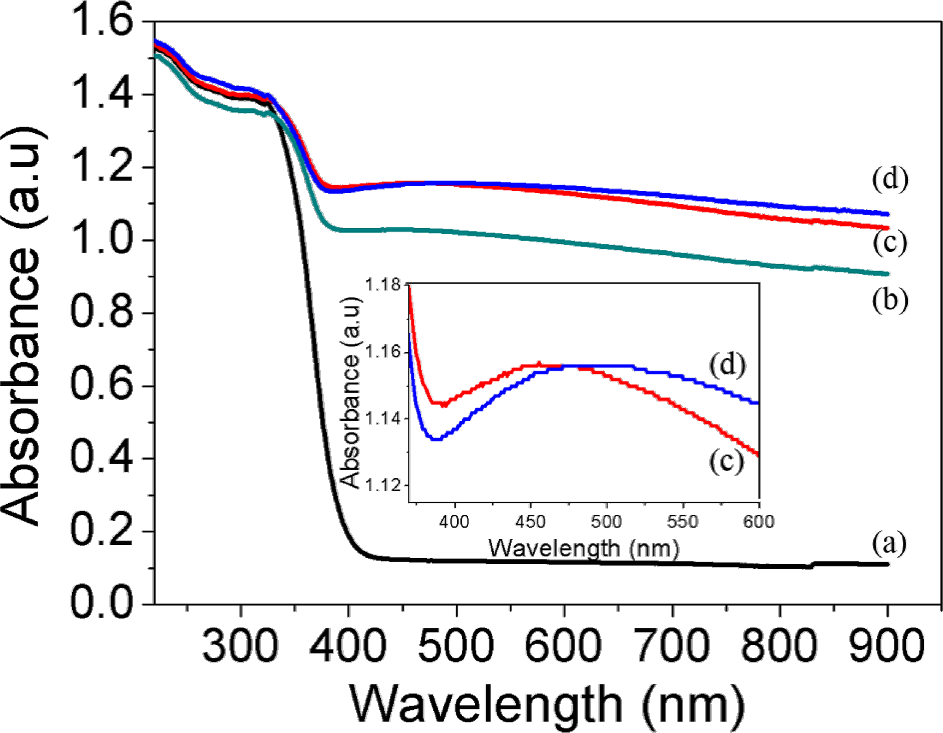
UV–vis absorption spectra of a) TiO_2_, b) 3.0 wt % Pd/TiO_2_, c) 0.5 wt % Pd/TiO_2_ and d) 1.0 wt % Pd/TiO_2_.

**Figure 8:**
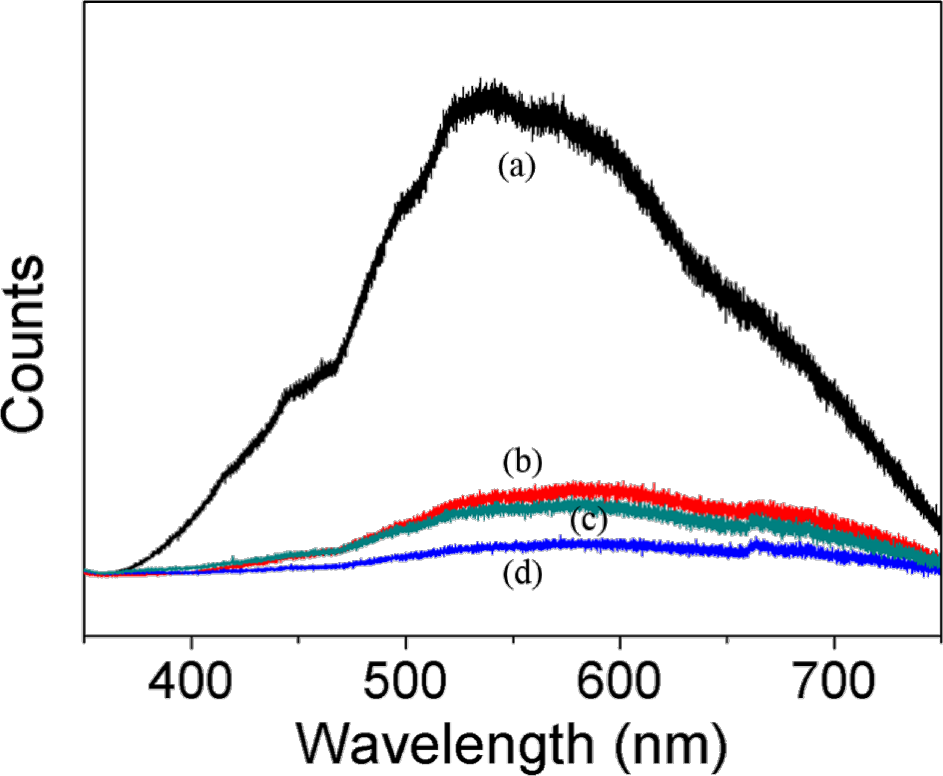
Photoluminescence spectra of a) TiO_2_, b) 0.5 wt % Pd/TiO_2_, c) 3.0 wt % Pd/TiO_2_ and d) 1.0 wt % Pd/TiO_2_.

The optical absorbance spectrum was calculated by using the UV–vis diffuse reflectance spectra and it is shown in Figure 7. As predicted and according to theory, the absorption band shown by anatase TiO_2_ sample due to the charge-transfer absorption from the oxide anions 2p orbital valence band to the conduction band of 3d orbital of Ti^4+^ cations is below 400 nm [3,54–55]. Furthermore, anatase TiO_2_ showed almost zero absorption in the visible region as indicated in the spectrum. However, the deposition of Pd NPs caused a significant increase of the absorption in the visible region because of the surface plasmon absorption of palladium particles. Pd particles smaller than 10nm are only able to absorb in the UV region, however larger and cluster particles will exhibit a red shift and an enhanced ability to absorb visible light [28,49]. This arises from the different polarization field induced through the surface charges affected by the amplitude and relative phase of the scattered and incident fields [56]. Thus, it correlates well with our present findings, in which the average size of palladium particles was in the range of 17–29 nm. Therefore, the broad absorption peak between 450–500 nm observed on Pd/TiO_2_ with different amounts of palladium clearly shows the contribution of metallic Pd NPs. Although a small amount of Pd NPs enhanced the visible light absorption, a further increase of the Pd loading leads to an excessive absorption. This induced light scattering phenomena that resulted in deprived performance [29,57]. PL spectra are often employed to understand the surface processes involving the photogenerated electron–hole pairs [58]. The obtained PL spectrum in Figure 8 shows the emission intensity that relates with the recombination rate of the excited electron and hole pairs. Lower PL intensity indicates a lower recombination rate due to more electrons being transferred or trapped. The emission peak of Pd/TiO_2_ is obviously quenched as compared to that TiO_2_. This further revealed that the deposition of Pd NPs has enhanced the trapping or transfer of electrons thereby suppressing the recombination. This high charge carrier separation efficiency extends the reactive electron–hole lifetime and, hence, leads to a better photocatalytic performance by Pd/TiO_2_ photocatalyst.

### Photocatalytic studies

**Figure 9:**
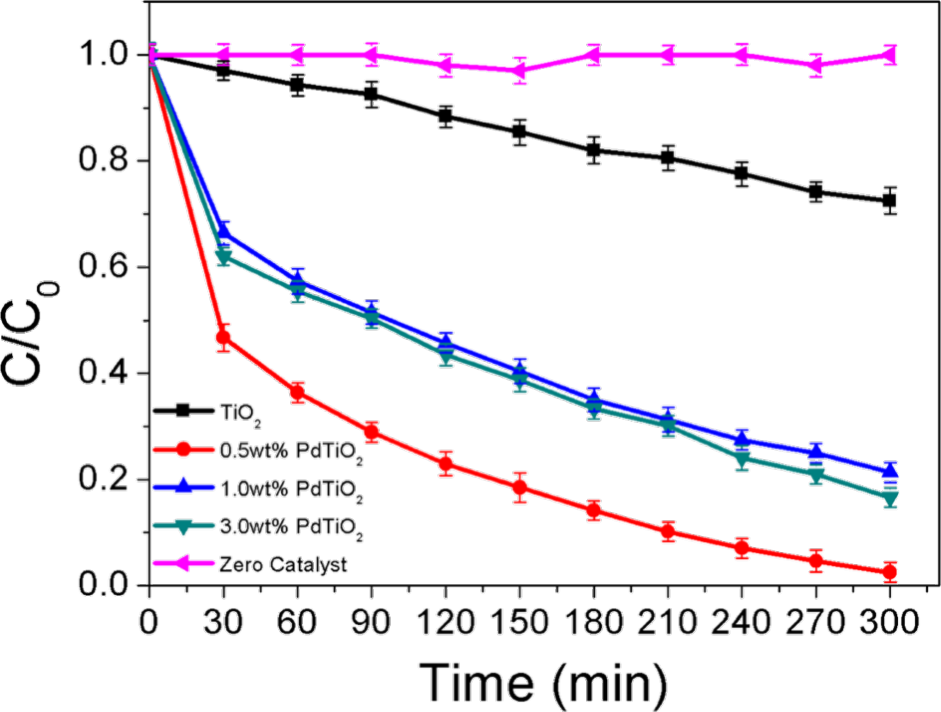
Photocatalytic degradation rates of AMX under visible light irradiation.

Figure 9 shows the photocatalytic performance of the prepared samples (Pd/TiO_2_) for the degradation of AMX under artificial visible light irradiation. The obtained results showed an excellent efficiency achieved irrespective of the palladium loading with an initial concentration of 20mg/L of AMX. From the experiment, the degradation efficiency of AMX followed an order of 97.5% (0.5 wt % Pd/TiO_2_) > 83.4% (3.0 wt % PdTiO_2_) > 78.7% (1.0 wt % PdTiO_2_) > 27.5% (TiO_2_) respectively. As expected the control experiment with the absence of photocatalysts showed almost no degradation of AMX. This proved the poor photolysis of AMX in the absence of photocatalysts. A significant enhancement in the degradation efficiency was achieved by depositing Pd NPs on the surface of TiO_2_. This immense progress was attributed to the localized surface plasmon resonance that enables Pd NPs to absorb light in the visible region. This is attributed to an optical excitation that produces a coherent oscillation of free electrons in resonance with the electrical field component of incoming electromagnetic irradiation [9,11,59]. When photons are absorbed by noble metal NPs, the electron density of the metal is polarized and oscillates resonantly at the light frequency [60]. The absorption of visible light by Pd NPs is also a function of the particle size. Smaller Pd particles (smaller than 10 nm) resonate well with UV irradiation, however larger particles and clusters resonate in the visible-light region [28,49,61]. The findings were observed in the present study in which a greater degradation was achieved for the Pd particles with a size of 17–29 nm. On the other hand, pure anatase TiO_2_ showed a very poor degradation of AMX (27.5%) after the same time. This was attributed to its own characteristic of having large band gap energy which hinder the absorption in the visible region as shown in our optical absorbance spectrum in which only a minimal absorbance was detected for TiO_2_. Therefore, only few electrons can be excited from the VB to the CB of TiO_2_, which leads to a low degradation efficiency.

**Figure 10:**
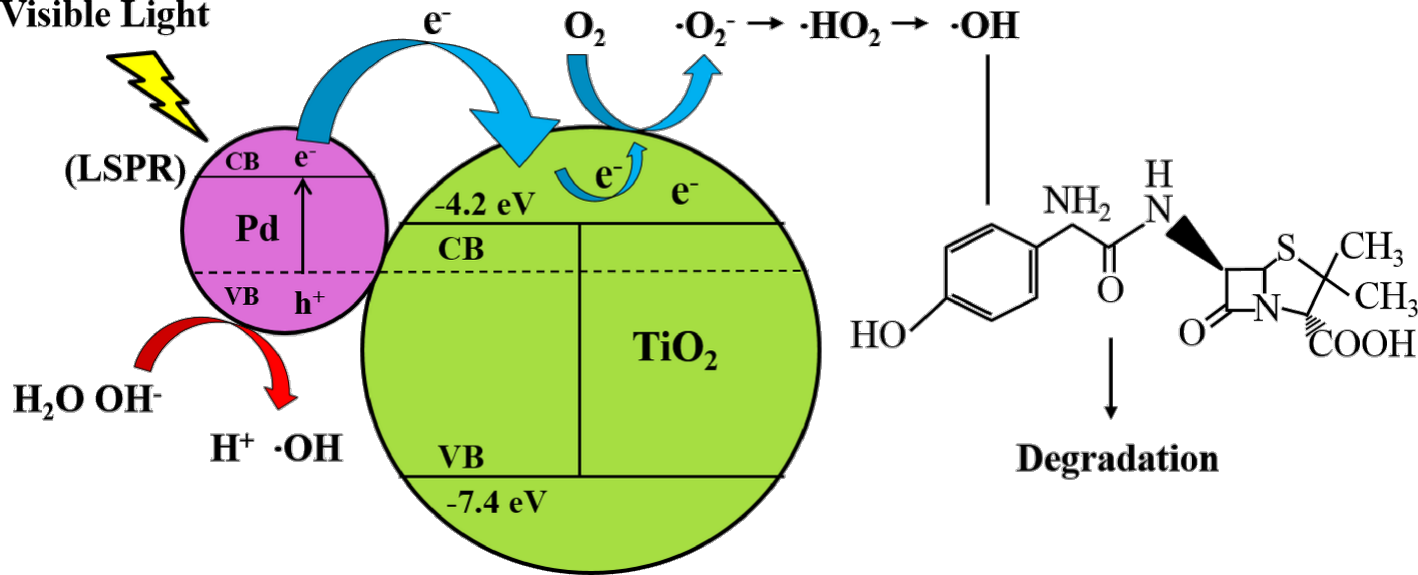
Schematic diagram of electron transfer and degradation mechanism of AMX.

The degradation mechanism and electron transfer is explained in Figure 10. When Pd/TiO_2_ is exposed to visible light, the plasmon resonance excites the electrons below the Fermi level of the Pd NPs in the VB to be transferred into the CB leaving behind the positive charges (h^+^) in the VB. As the CB of TiO_2_ is an electron acceptor, it readily accepts the electrons that are transferred from the Pd NPs to form superoxide anion radicals (•O_2_^−^). This is followed by protonation that yields •HO_2_ radicals. These instable •HO_2_ radicals further form H_2_O_2_ and lead to the formation of hydroxyl radicals (•OH), an active species that is responsible for the degradation of AMX. Besides that, when Pd NPs interacts with TiO_2_, it will form a Schottky barrier at which an internal electric field close to the interface is generated. Hence, it will drive the electrons and holes to move in different directions and the photon energies of electrons excited upon LSPR excitation is able to cross the energy junction at interface. As a result electrons are transferred from Pd NPs to the CB of TiO_2_ [9,62]. Furthermore with the formation of the Schottky barrier, the lifetime of the charge carriers is increased [63]. This mechanism is illustrated in Figure 10.

Meanwhile, the study of the influence of the palladium weight percentage (0.5 wt %, 1.0 wt % and 3.0 wt %) on the photocatalytic efficiency showed an optimum value at 0.5 wt %. According to the optical spectra and photoluminescence analysis the 1.0 wt % Pd/TiO_2_ was expected to yield a better photocatalytic efficiency than the others. This unexpected observance can be attributed to the following reasons. During the irradiation of the photocatalyst particles photons will be absorbed and scattered. As observed in the optical spectra, a peak broadening phenomenon was observed for 1.0 wt % owing to light scattering effects. Thus the absorption of visible light is reduced due to the shielding effect by the Pd layers, which results in less active electrons being generated [29,57].

**Figure 11:**
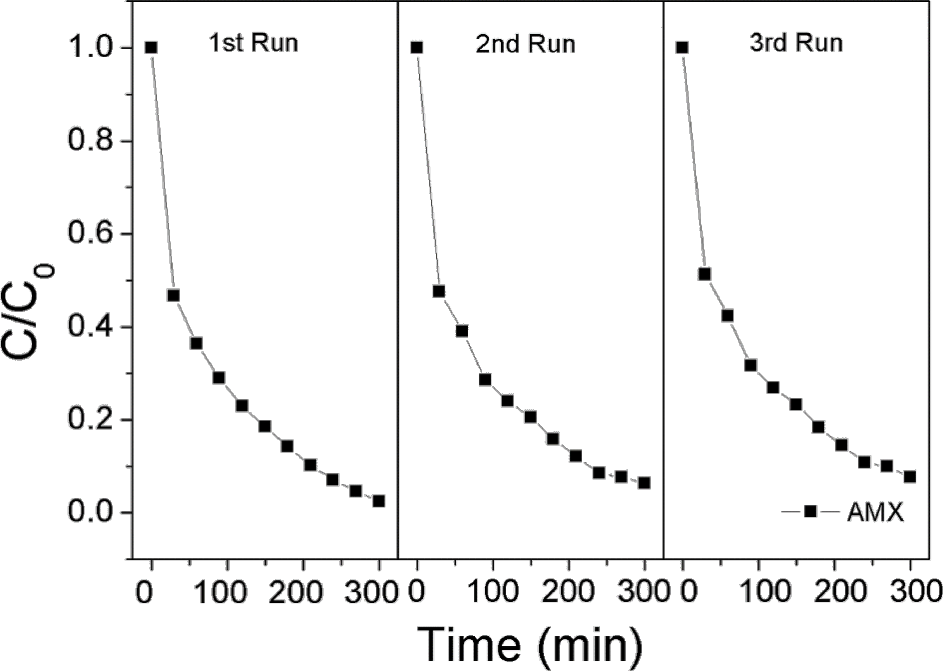
Recycled photocatalytic degradation rates of AMX (0.5 wt % Pd/TiO_2_).

**Figure 12:**
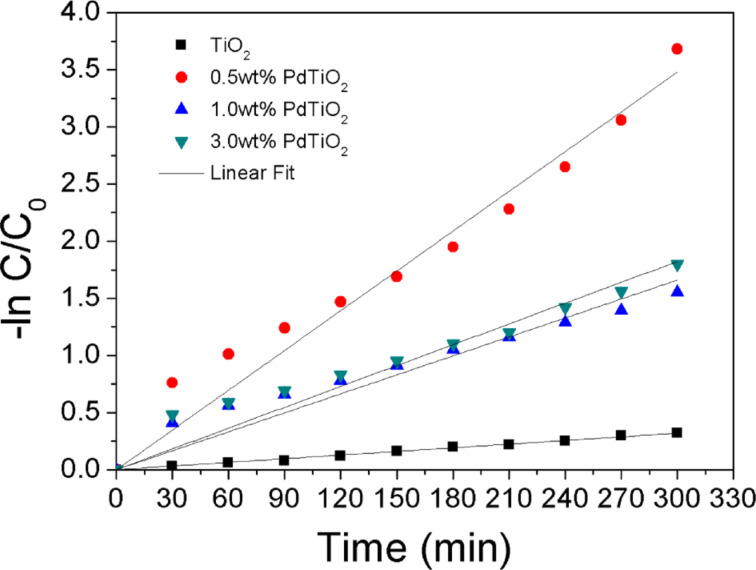
The kinetics of AMX degradation by prepared TiO_2_ and various Pd loading photocatalysts.

The stability of the prepared photocatalyst is important for practical applications. Therefore a recycle experiment was carried out under identical conditions. As shown in Figure 11, the photocatalytic activity of the as prepared 0.5 wt % Pd/TiO_2_ photocatalyst maintains a high level of degradation efficiency after three times of recycling. An efficiency of 92.3% was achieved after the 3rd run, which indicates an excellent photostability of the synthesized photocatalyst. The kinetics of the photocatalytic degradation of AMX are of pseudo-first-order (Figure 12). The obtained kinetics parameters are tabulated in Table S2 (Supporting Information File 1). Finally, the degree of mineralization of AMX during the photocatalytic degradation was also presented through a total organic carbon (TOC) analysis and depicted in Figure S3 (Supporting Information File 1).

## Conclusion

The present study shows the successful synthesis of the heterogeneous photocatalyst Pd/TiO_2_ through a smart, easy and sustainable approach. The synthesized TiO_2_ exhibited exclusively the anatase phase. The natural light led to the reduction and anchoring of Pd NPs onto the surface of TiO_2_, and the large size of the Pd NPs contributed to the visible-light absorption. Localized surface plasmon resonance (LSPR) and the formation of a Schottky barrier at the TiO_2_ interface also occured. The prepared heterogeneous photocatalysts exhibited superior photocatalytic activity and stability under artificial visible light irradiation by almost completely degrading AMX after a short time. The absorption over the full visible region with high photoactivity and stability laid a pathway for future practical sustainable applications. Thus, the present study is a significant contribution towards non-renewable energy sources by synthesizing a visible-light photocatalyst for sustainable applications.

## Experimental

### Materials

Titanium(IV) chloride (TiCl_4_ 99.9%, Merck), sodium tetrachloropalladate(II) (Na_2_[PdCl_4_] 99.998%, Sigma–Aldrich), tetrahydrofuran (THF, Fluka), ethylene glycol and benzyl alcohol (99.8% anhydrous, R&M Chemicals) and deionized water. All chemicals were analytical grade and used as received without any further purification.

### Synthesis of anatase TiO_2_ NPs

TiCl_4_ (1 mL) was added dropwise into 20 mL of anhydrous benzyl alcohol under vigorous stirring in a controlled inert gas (nitrogen) atmosphere. After complete addition, a yellowish solution with white precipitate at the bottom of the beaker is obtained. The solution was then aged for 21 days at room temperature. The resulting white precipitate after aging was recovered by centrifugation at 3500 rpm for 15 min followed by washing with 20 mL of ethanol and tetrahydrofuran (THF) for few times. After washing, the obtained TiO_2_ white powder was dried at room temperature and grind into powder. The grind sample was then calcined at 450 °C for 4 h.

### Synthesis of Pd/TiO_2_ NPs

The prepared anatase TiO_2_ (0.4 g) was added to 12 mL of ethylene glycol containing a chosen amount of Na_2_PdCl_4_ (0.5 wt %, 1.0 wt % and 3.0 wt %). Thus the above chosen precursors weight percentage yielded 0.5 wt % Pd, 1.0 wt % Pd and 3.0 wt % Pd loading, respectively. Then the mixture was subjected to a sustainable photodeposition through a reduction reaction excited under sunlight with intensities between 150 and 180 W·m^−2^ for 30 min. The obtained precipitate was recovered by centrifugation at 2000 rpm for 5 min. Thus obtained Pd/TiO_2_ was then washed repeatedly with ethanol and deionized water and dried overnight at 90 °C.

### Characterization

The morphology of the samples were investigated by field emission scanning electron microscopy (FESEM, Hitachi SU-8000) equipped with an energy dispersive X-ray spectrometer (EDS, Zeiss Auriga). The images were taken at an accelerating voltage of 20 kV. High resolution transmission electron microscope (HRTEM, JEM-2100F, Jeol) images were obtained at 200 kV. The phase composition of the prepared photocatalysts was analyzed by X-ray diffraction (XRD, Bruker D8 advance X-ray powder diffractometer with Cu Kα radiation λ = 0.154 nm). A micro-PL/Raman spectroscope (Renishaw, inVia Raman Microscope) was used to acquire the Raman and photoluminescence (PL) spectra with the excitation wavelengths of 514 and 325 nm, respectively. Brunauer–Emmett–Teller (BET) surface area, pore volume, and Barret–Joyner–Halenda (BJH) pore size distribution based on nitrogen adsorption–desorption isotherms were analyzed with a TriStar II 3020 (Micrometrics^®^, USA) surface area and porosity system. Prior to the analysis, the samples were degassed at 150 °C for 5 h under nitrogen atmosphere. X-ray photoelectron spectra (XPS) were obtained with Axis Ultra DLD instrument of Kratos by using monochromatic Al Kα radiation (225 W, 15 mA, 15 kV). The C1s binding energy of adventitious carbon (284.9 eV) was used as reference. UV–vis diffuse reflectance spectra (UV-DRS) were performed with a Shimadzu UV-2600 spectrophotometer equipped with an integrating sphere attachment. The spectra were obtained with BaSO_4_ as a reference.

### Photocatalytic activity

The photocatalytic activity of the prepared samples was evaluated by degrading amoxicillin. These experiments were carried out in a batch process in a simple 500 mL borosilicate beaker with 250 mL working volume (initial concentration of amoxicillin = 20 mg/L) under stirring by adding 1 g of the synthesized photocatalysts. A 500 W tungsten-halogen lamp was used as visible light source with a high pass UV light filter (FSQ-GG400, Newport Corp.). All experiments were carried out under identical conditions. The control experiment was carried out without photocatalyst to ensure the degradation was solely due to the presence of the photocatalyst. The samples were drawn at regular intervals and the concentration of amoxicillin was measured by using an ultra-performance liquid chromatography (UPLC) equipped with UV–visible detector (Acquity UPLC H-Class, Waters). The UPLC was mounted with a C18 column at 40 °C (2.1 × 50 mm, 1.7 μm particle size) using KH_2_PO_4_ (pH 1.8)/methanol (80:20) as mobile phase at a flow rate of 0.4 mL/min. The amoxicillin was detected with a set wavelength of 228 nm. The degrees of mineralization of AMX were appraised from the amount of total organic carbon (TOC) using O.I Analytical Aurora 1030W TOC Analyzer. All photocatalytic experiments were carried out for 5 h.

## Supporting Information

File 1 Additional experimental data.
