# Supplementary material for: Palladium nanoparticles anchored to anatase TiO2 for enhanced surface plasmon resonance-stimulated, visible-light-driven photocatalytic activity
Source: Beilstein J Nanotechnol. 2015 Feb 11;6:428–37. doi: 10.3762/bjnano.6.43 (PMC4362308; doi:10.3762/bjnano.6.43)
Supplement: File 1 — Additional experimental data. [file Beilstein_J_Nanotechnol-06-428-s001.pdf]

Supporting Information

for

Palladium nanoparticles anchored to anatase TiO_2 for enhanced surface plasmon resonance-stimulated, visible-light-driven photocatalytic activity

Kah Hon Leong[1], Hong Ye Chu[1], Shaliza Ibrahim[1] and Pichiah Saravanan*[1,2]

Address: [1]Environmental Engineering Laboratory, Department of Civil Engineering, Faculty of Engineering, University of Malaya, 50603, Kuala Lumpur, Malaysia and [2]Nanotechnology & Catalysis Research Center (NANOCAT), University of Malaya, 50603, Kuala Lumpur, Malaysia

Email: Pichiah Saravanan* - saravananpichiah@um.edu.my

*Corresponding author

Additional experimental data

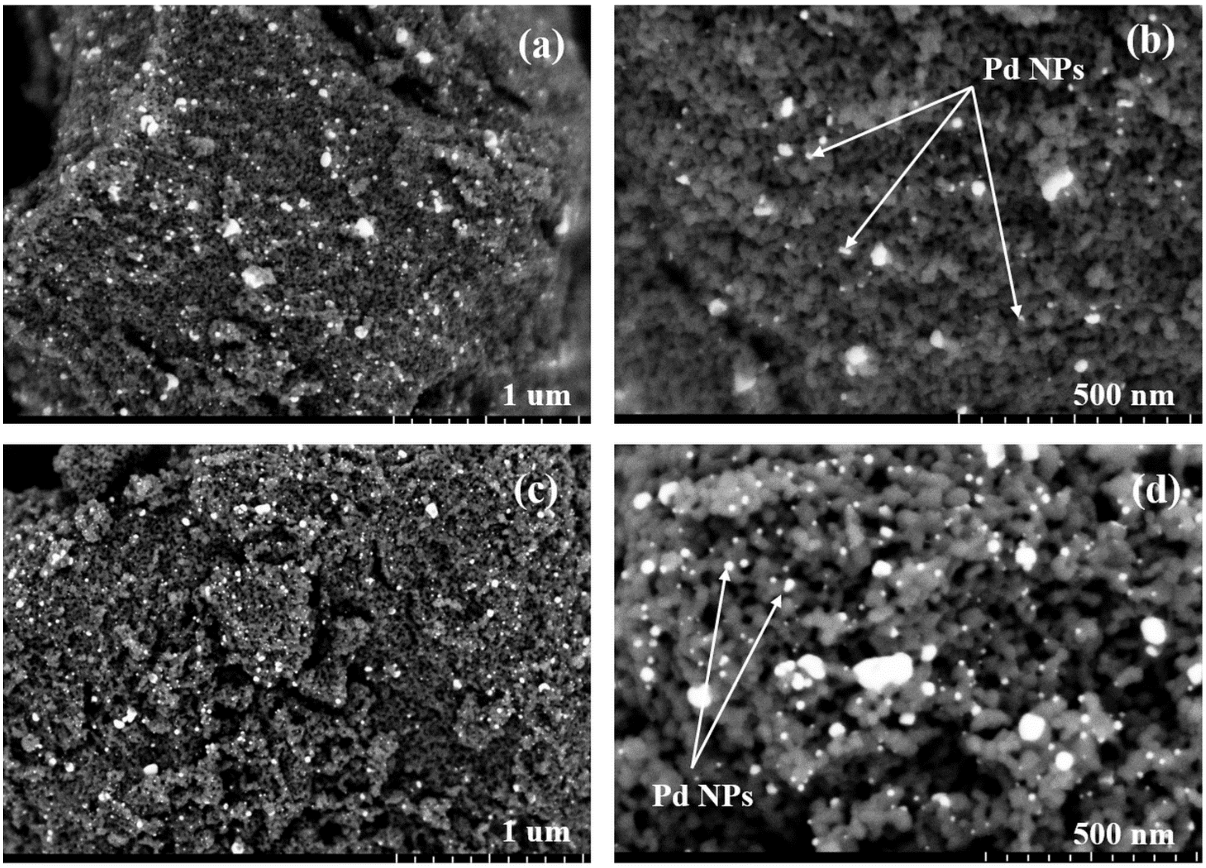

Figure S1: FESEM images of (a,b) 1.0 wt % Pd/TiO_2 and (c,d) 3.0 wt % Pd/TiO_2.

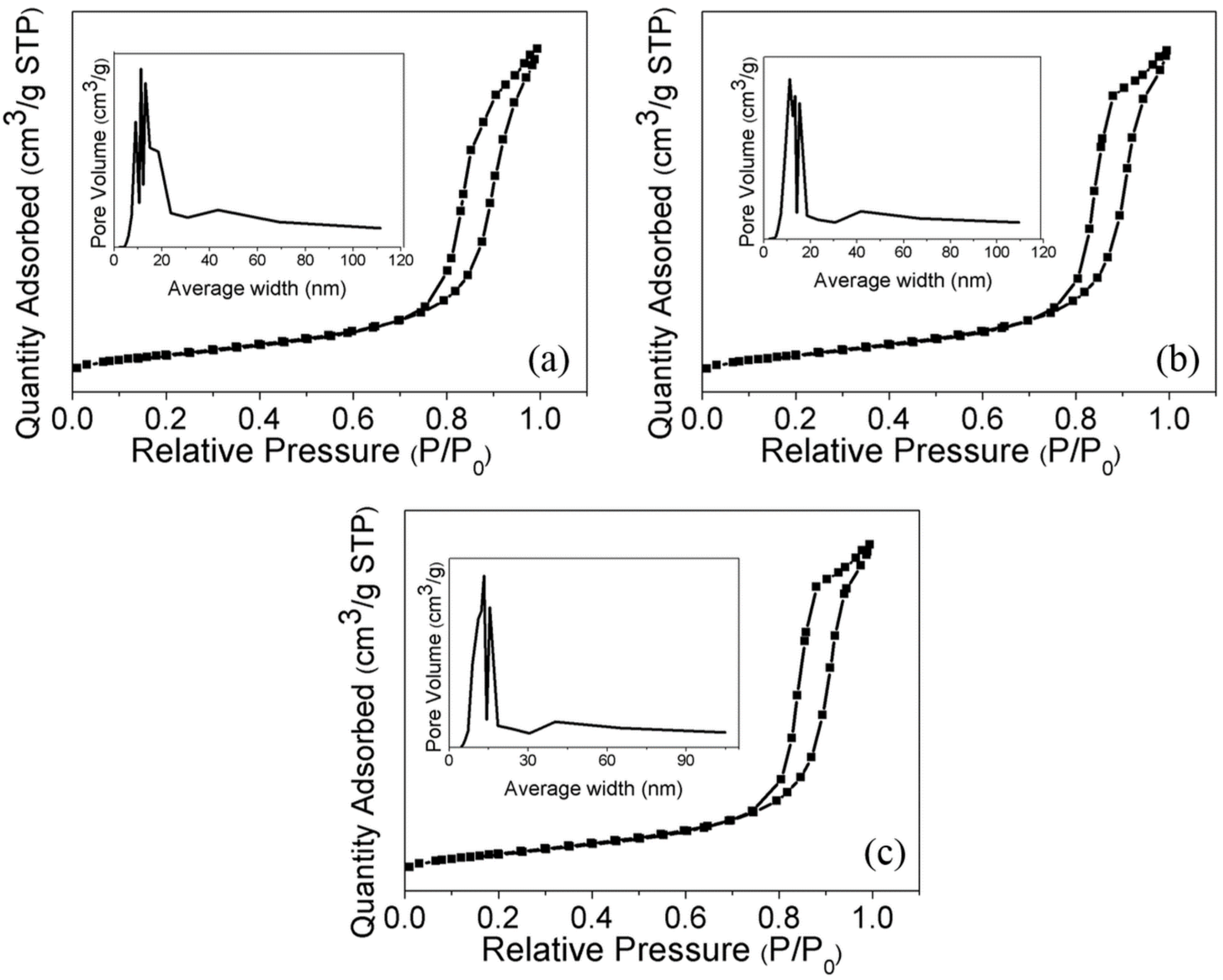

Figure S2: Adsorption–desorption isotherms of (a) TiO_2, (b) 1.0 wt % Pd/TiO_2, (c) 3.0 wt % Pd/TiO_2 and the inset is the pore size distribution.

Table S1: The BET surface area, average pore size and pore volume of TiO_2 and different Pd loadings.

	**BET surface area (m^2/g)**	**Average pore size (nm)**	**Pore volume (cm^3/g)**
anatase TiO_2	52.2374	12.9256	0.2098
0.5 wt % Pd/TiO_2	46.6761	14.3101	0.2031
1.0 wt % Pd/TiO_2	48.7703	12.9295	0.1949
3.0 wt % Pd/TiO_2	48.6139	13.0052	0.1978

Table S2: Photocatalytic degradation kinetic parameters of AMX for TiO_2, 0.5 wt % Pd/TiO_2, 1.0 wt % Pd/TiO_2 and 3.0 wt % Pd/TiO_2.

	First Order Kinetics			
	TiO_2	0.5 wt % Pd/TiO_2	1.0 wt % Pd/TiO_2	3.0 wt % Pd/TiO_2
R^2	0.9983	0.9901	0.9811	0.9845
k	0.00107	0.01161	0.00553	0.00608

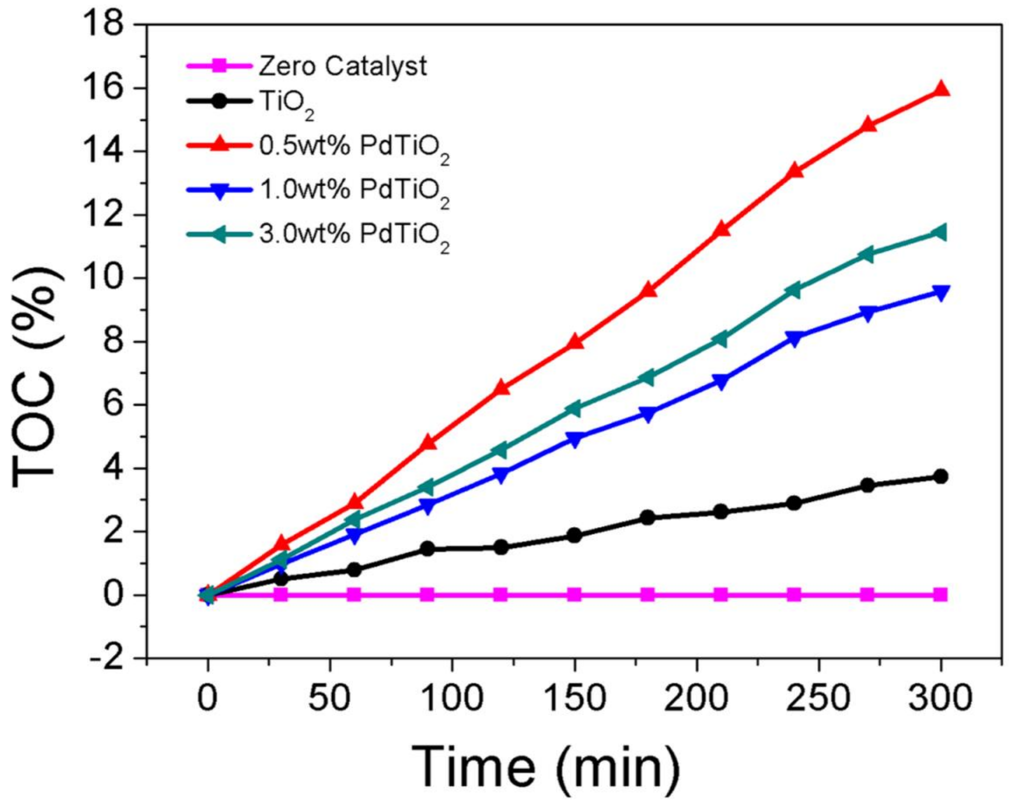

Figure S3: Percentage mineralization of AMX.
